# Validation of a Heart Failure Risk Score in a Cohort of Cardiac Resynchronization Therapy Patients Under Remote Monitoring: Results from the TriageHF™ Algorithm

**DOI:** 10.19102/icrm.2023.14093

**Published:** 2023-09-15

**Authors:** Isabel Cardoso, Pedro Silva Cunha, Sérgio Laranjo, André Grazina, José Miguel Viegas, Guilherme Portugal, Bruno Valente, Ana Lousinha, Pedro Brás, Manuel Brás, Rui C. Ferreira, Mário Oliveira

**Affiliations:** ^1^Cardiology Service, Central Lisbon Hospital and University Centre, Lisbon, Portugal; ^2^Arrhythmology, Pacing, and Electrophysiology Unit, Cardiology Service, Central Lisbon Hospital and University Centre, Lisbon, Portugal; ^3^Institute of Physiology and CCUL, Faculty of Medicine of Lisbon, Lisbon, Portugal; ^4^Cardiology Center, Hospital CUF Tejo, Lisbon, Portugal

**Keywords:** Cardiac resynchronization therapy defibrillators, heart failure, heart failure risk status, HF-related hospitalizations, remote monitoring, TriageHF™

## Abstract

The heart failure risk status (HFRS) is a validated dynamic tool for risk score prediction, based on the TriageHF™ algorithm (Medtronic, Minneapolis, MN, USA), for the occurrence of a heart failure (HF) event in the 30 days following a remote monitoring (RM) transmission. The aim of this study was to evaluate the accuracy of the HFRS in predicting an unplanned hospital admission due to HF decompensation in a real-world cohort of patients submitted to cardiac resynchronization therapy (CRT). We conducted a single-center review of a cohort of 40 consecutive HF patients, under RM, with CRT devices using the HFRS of the TriageHF™ algorithm. The correlation of the HFRS with hospital admissions was analyzed. During a mean follow-up of 36 months, a stepwise increase in the HFRS was significantly associated with a higher risk of HF admission (odds ratio, 12.7; 95% confidence interval, 3.2–51.5; *P* < .001), and the HFRS was demonstrated to have good discrimination for HF hospitalization, with an area under the receiver-operating characteristic curve of 0.812. The TriageHF™ algorithm effectively predicted HF-related hospitalization in a cohort of CRT patients during long-term RM follow-up, providing a novel clinical pathway to optimize the clinical management of this complex population.

## Introduction

Hospitalizations due to heart failure (HF) are common, with a negative impact on the quality of life and patient prognosis and driving growing health care costs.^1–3^ In recent years, there has been growing interest in identifying novel strategies for the prediction of HF decompensation, mostly based on remote monitoring (RM) programs. The early detection of an increased risk for decompensation is challenging but opens an opportunity for the health care team to have a positive impact on the outcomes of this population.

The RM of HF patients with cardiac implantable electronic devices (CIED) has become a standard of care.^4^ The RM follow-up not only allows monitoring of CIED functions but also provides an individualized dynamic evaluation of various physiological parameters, such as thoracic impedance, arrhythmia burden, percentage of pacing, night heart rate (NHR), heart rate variability (HRV), or patient activity levels over time. Information is transferred on a regular basis, and alerts are forwarded to the clinicians.^5^ Some routinely monitored parameters may reflect the patient’s clinical status and predict impending cardiac decompensation.^6^ Over recent years, the impact of RM on clinical outcomes for patients with HF has had variable results in different randomized trials, and therefore more real-world data are needed to clarify the potential utility of RM in this population.^7–12^

The HF risk status (HFRS) is a validated dynamic HF risk-prediction tool, available on CIEDs from Medtronic (Minneapolis, MN, USA), that integrates diagnostic data to generate a patient-specific assessment of “low,” “medium,” or “high” risk score for HF hospitalization in the 30 days after each data-transmission episode.^13,14^ The TRIAGE-HF trial prospectively studied the HFRS and found that a high HFRS has good predictive accuracy for HF decompensation.^5^ In the present study, we aim to assess the value of the HFRS algorithm in predicting unplanned hospital admissions of real-world HF patients submitted to cardiac resynchronization therapy (CRT).

## Materials and methods

### Study population

We conducted a retrospective review of HF patients aged >18 years, with reduced left ventricular ejection fraction (LVEF), under optimal medical therapy who underwent HFRS-enabled Medtronic CRT device implantation and had ≥12 months of RM follow-up between January 2014 and September 2019.

The study was conducted in accordance with the Declaration of Helsinki, and each participant provided informed consent prior to participation.

Collection and detailed analysis of all data available for the study population regarding RM transmissions with the HFRS information was performed. Data on baseline information, hospital admissions, and cardiovascular and all-cause events were collected through chart reviews and by phone contact.

### Study design

Demographic data, clinical outcomes, and the data extracted from the Medtronic CareLink™ network were obtained. Patients were monitored via a monthly automated remote download regimen, with a scheduled biannual face-to-face outpatient clinic visit. The HFRS given in each transmission reflected the highest daily score in the preceding 30 days. Patients were classified into low-, medium-, and high-risk groups according to the HFRS.^13^ Device diagnostic parameters, including thoracic impedance (OptiVol^®^; Medtronic), patient activity, NHR, HRV, percentage of biventricular pacing, atrial tachycardia/atrial fibrillation (AT/AF) burden, ventricular rate during AT/AF (VRAF), and detected arrhythmia episodes/therapy delivered, were classified such that a lower level (ie, level 1) indicated normal values and higher levels implied increasingly abnormal values. The definition and classification of each were as follows:

1. Intrathoracic impedance was quantified across the right ventricular coil and device by injecting a small current pulse and measuring the developed voltage. The OptiVol^®^ index was computed as the accumulation of the difference between the daily and the reference thoracic impedance and classified into 4 levels (level 1, <30 Ω/day; level 2, 30 to <60 Ω/day; level 3, 60–100 Ω/day; level 4, >100 Ω/day).
2. Activity was measured by a single-axis accelerometer in the device and reported as active minutes per day (level 1, >60 min/day; level 2, ≤60 min/day or decreasing trend).
3. NHR, HRV, AF burden, and VRAF were derived from atrial and ventricular electrograms acquired via the CIED. NHR was the average heart rate between midnight and 4 a.m. and is a measure of the resting heart rate (level 1, 55–85 bpm; level 2, ≥85 or ≤55 bpm or increasing trend).
4. HRV was measured as the standard deviation of sinus rhythm intervals during a 24-h period (level 1, >60 ms; level 2, ≤60 ms or decreasing trend).
5. AT/AF burden was measured as the total duration of the fast atrial rate during a 24-h period (level 1, <60 min/day; level 2, ≥60 min/day).
6. VRAF is the average ventricular rate during AF over a 24-h duration and was considered abnormal (level 2) if the ventricular rate was ≥90 bpm and the AF burden was ≥6 h/day.

These parameters were integrated using a Bayesian belief network approach to compute a numeric score ranging from 0–1 point(s). A risk score of <0.054 points was categorized as low HFRS, that between 0.054–0.20 points was categorized as medium HFRS, and that of ≥0.20 points was categorized as high HFRS. In a previous study, a low HFRS was associated with an HF hospitalization rate of 0.6% in the next 30 days, a medium HFRS was associated with an HF hospitalization rate of 1.3%, and a high HFRS was associated with an HF hospitalization rate of 6.8%, respectively.^5^

In addition to the HFRS data received, patient-activated transmissions in the presence of symptoms were also recorded, and the “Care Alert” function was automatically activated in response to the detection of AF episodes, ventricular arrhythmias, or high OptiVol^®^. Hospital admissions were systematically assessed by the use of a national database (“Plataforma de Dados de Saúde”).

### Endpoints

The primary objective of this study was to evaluate the accuracy of the HFRS algorithm in the prediction of unplanned hospital admission due to HF decompensation in a real-world cohort of patients submitted to CRT. The secondary outcomes included the changes in the different clinical variables—namely, thoracic impedance (OptiVol^®^), patient activity, NHR, HRV, percentage of biventricular pacing, AT/AF burden, VRAF, and detected arrhythmia episodes/therapy delivered.

### Statistical analysis

Standard descriptive statistics were used to describe the clinical characteristics within the patient groups. Continuous parameters were presented as mean and standard deviation values when they followed a normal distribution and as median and interquartile range values otherwise. Categorical variables were expressed using frequency and percentage values. Comparisons were performed using the chi-squared test for qualitative data and Student’s *t* test for continuous variables. The accuracy of the HFRS algorithm was evaluated by means of random-effects logistic regression for the outcome of unplanned hospital admission for HF in the 30 days following each transmission episode. The analysis of the primary outcome of unplanned hospital admission for HF was performed using Kaplan–Meier curves, the log-rank test was considered for comparisons, and a Cox proportional hazards model was used for calculation of the hazard ratio. A two-sided *P* value of <.05 was considered statistically significant. Data were managed and analyzed by using the SPSS Statistics program (version 24; IBM Corporation, Armonk, NY, USA).

## Results

### Patient demographics

A total of 40 patients were included, with a mean follow-up period of 36 ± 11 months. **Table 1** summarizes the clinical and demographic characteristics of the studied population.

**Figure 1:**
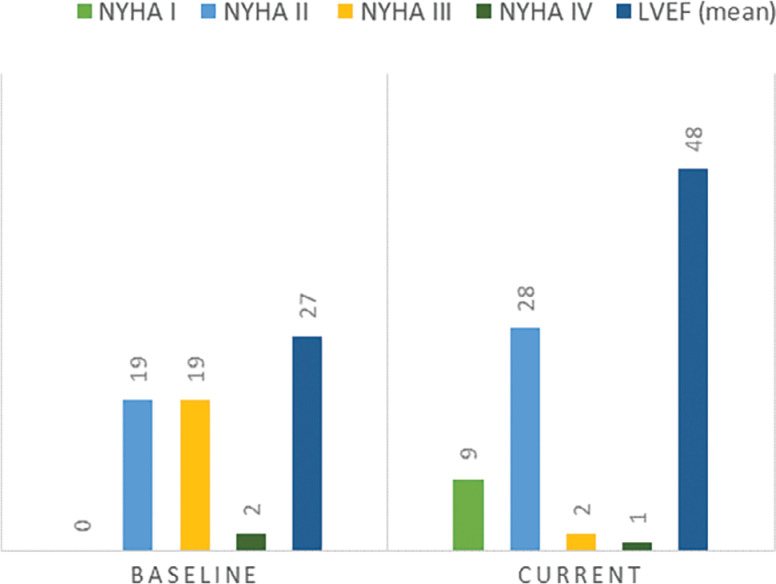
Progression of left ventricular ejection fraction and New York Heart Association class during the follow-up period.

During the follow-up period, CRT resulted in significant improvements in LVEF (27% ± 8% vs. 48% ± 12%, *P* < .001) and New York Heart Association (NYHA) class > II (51% vs. 18%, *P* = .006) **(Figure 1)**. The clinical response rate based on NYHA class and ejection fraction rate was 85%.

### Transmissions

**Figure 2:**
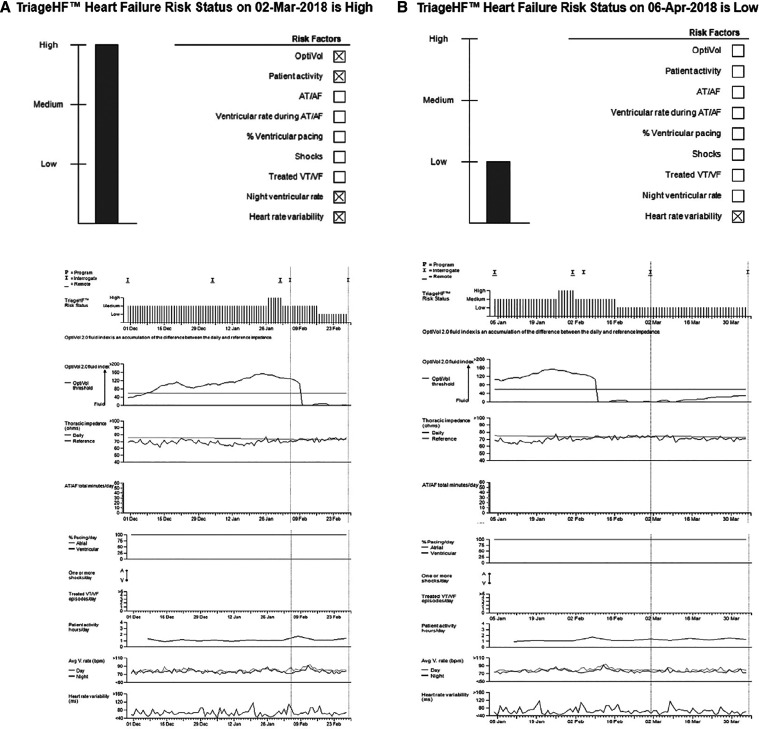
Example of a patient with abnormal heart failure risk status (HFRS) parameters leading to a high-risk HFRS score **(A)** with subsequent improvement to a low-risk HFRS score **(B)**. *Abbreviations:* AT/AF, atrial tachycardia/atrial fibrillation; VT/VF, ventricular tachycardia/ventricular fibrillation.

A total of 1108 transmissions with HFRS data of all patients corresponding to 94 patient-years were collected. Of these transmissions, 376 (34%) were low-risk HFRS transmissions, 633 (57%) were medium-risk HFRS transmissions, and 99 (9%) were high-risk HFRS transmissions, respectively. In 20% of the cases, there was ≥1 high-risk score transmission. The most common abnormal HFRS parameters identified in the collected transmissions were decreased HRV (44%), abnormal NVR (43%), and reduced patient activity (34%). OptiVol^®^ was abnormal in 19%, the percentage of biventricular pacing was 16%, the AT/AF burden was 4%, arrhythmic episodes or delivered therapies affected 0.6%, and VRAF was present 0.5% of the transmissions, respectively **(Figure 2)**.

### Events

**Figure 3:**
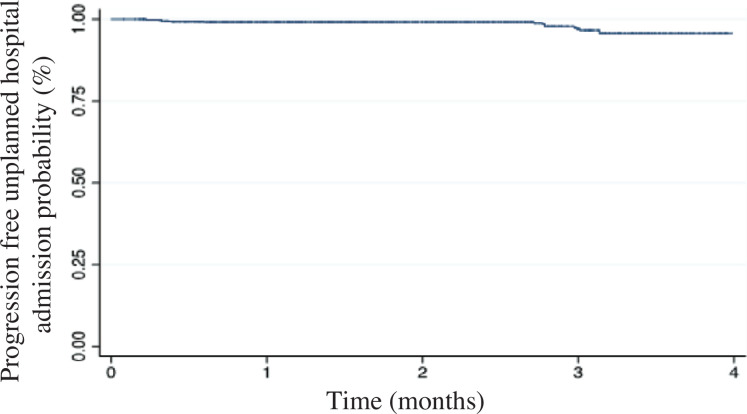
Freedom from unplanned hospital admission (n = 40). Unplanned hospital admissions due to HF decompensation were observed in 6 patients (15%), corresponding to an event rate of 3.5%/year.

Unplanned hospital admissions due to HF decompensation were observed in 6 patients (15%), corresponding to an event rate of 3.5%/year **(Figure 3)**. Hospital admissions for HF were observed within 30 days after 9 data transmissions of different patients (6 of high risk and 3 of medium risk). A total of 7 device-appropriate therapies for sustained ventricular tachyarrhythmias (VT/VF) were delivered, corresponding to an annual event rate of 7.4%. No deaths occurred during the follow-up period.

**Figure 4:**
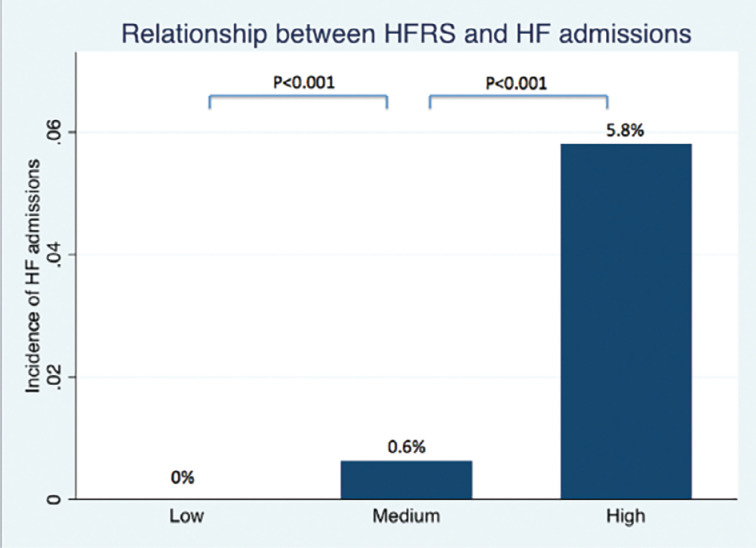
Relationship between heart failure risk status and heart failure admissions. A stepwise increase in heart failure risk status was significantly associated with a higher risk of heart failure admission (odds ratio, 12.7; 95% confidence interval, 3.2–51.5; *P* < .001).

Stepwise increase in HFRS was significantly associated with a higher risk of HF admission (odds ratio, 12.7; 95% confidence interval [CI], 3.2–51.5; *P* < .001) **(Figure 4)**. HFRS had good discrimination for HF events, with an area under the receiver-operating characteristic curve of 0.812.

## Discussion

In this study, we evaluated the performance of the TriageHF™ algorithm (Medtronic, Minneapolis, MN, USA) in predicting HF decompensation in a high-risk cohort of patients submitted to CRT implantation in a real-world population. We showed that HFRS can predict unplanned HF admissions.

The main findings of the study were as follows: (1) in an HF population submitted to successful CRT implantation, only 9% of transmissions showed a high HFRS during 36 months of follow-up; (2) the most common parameters associated with an increased HFRS were decreased HRV, abnormal ventricular rate at night, and reduced patient activity; (3) the HF event rate was considered low (3.5%/year), but a stepwise HFRS increase showed good discrimination in detecting HF decompensation, with an odds ratio of 12.7; and (4) low- and moderate-risk profiles were associated with a low risk of hospital admission.

Regarding patients with a medium-risk score, although a higher rate of hospitalization was verified compared to that of low-risk patients, more than half of the transmissions (57%) corresponded to a medium-risk score. Therefore, a medium-risk HFRS might have less clinical relevance when monitoring patients with HF.

The presence of a small number of HF events in this population suggests that CRT patients under an RM program have a potential lower risk for HF admissions. This can be, in part, attributed to a high proportion of CRT responders, possibly allowing up-titration of HF therapy to optimal doses, a significant reduction of patients with NYHA class > II, and a routine management of automated alerts (CareAlerts notifications).

The RM of HF patients with CIED has shown variable results in demonstrating significant improvement regarding HF hospitalization and survival.^3,15^ Clinical trials such as Remote Management of Heart Failure Using Implantable Electronic Devices (REM-HF) and Monitoring Resynchronization Devices and Cardiac Patients (MORE-CARE) failed to show an impact on hard outcomes.^2,5^ On the other hand, recent meta-analyses point to significant clinical benefits.^1,6,12^ The Implant-based Multiparameter Telemonitoring of Patients with Heart Failure trial showed improved clinical outcomes for HF patients with CIEDs, leading to the inclusion of a multiparameter telemonitoring approach in the European Society of Cardiology HF guidelines.^4,11,16^

The need for accurate and practical RM tools has been greater since the start of the coronavirus disease 2019 (COVID-19) pandemic. Stay-at-home orders and physical distancing have been essential to reduce the spread of severe acute respiratory syndrome coronavirus 2, especially early on. In this setting, an RM program using the HFRS is a promising tool to complement virtual visits during the COVID-19 pandemic. The large-scale real-world data are lacking, but the current COVID-19 pandemic context may justify larger trials looking for more robust evidence of clinical benefits in this setting.^13^

Our data are consistent with previous CIED studies using the TriageHF™ algorithm and show that, even in a CRT population with a high rate of responders and a low incidence of HF admissions, the HFRS has very good accuracy in predicting HF events. This translates into an individualized way of realizing that a particular patient is at risk of decompensation and helps us to follow HF patients more closely and safely, without the need for their presence. Studies looking into the impact on prognosis improvement and cost reduction are mandatory for the appropriate organization of care toward HF management.

### Study limitations

The present work has some limitations. This was a retrospective study and, therefore, unmeasured confounding factors may have influenced the observed associations. In addition, this was a single-center study with a relatively small sample size. Nevertheless, endpoints were predefined, and the CRT population (constituted mainly by responders) was well characterized. Also, despite the occurrence of a small number of HF-related events, it was possible to show the good accuracy of the HFRS algorithm to predict HF decompensation. Another limitation concerns the fact that transient shifts in one parameter can trigger a high HFRS, without any true clinical consequence. In fact, all transmissions received during the study period were taken into account (even if in the same week or day), which can potentially amplify this weak point. Finally, the results refer to devices with the TriageHF™ algorithm from Medtronic and, therefore, may not apply to similar scores derived from other manufacturers.

## Conclusions

The TriageHF™ algorithm effectively predicted HF-related hospitalization in a cohort of high-rate responders to CRT during a long-term RM follow-up, providing a novel clinical pathway to optimize the health management of this population with complex needs. Future research should determine whether this integrated data management tool may improve outcomes in the HF population by actively treating high-risk patients.

## Figures and Tables

**Table 1:** Patient Demographics

	Total
Patients, n (%)	40
Age, years (mean ± SD)	72 ± 10
HF etiology, n (%)	
Dilated cardiomyopathy	21 (52.5)
Ischemic heart disease	15 (37.5)
Valvular heart disease	4 (10)
Ejection fraction (implantation), % (mean ± SD)	27 ± 8
Clinical responder rate, %	85
NYHA class at implantation, n (%)	
II	19 (47.5)
III	19 (47.5)
IV	2 (5)
BNP at implantation, pg/mL (mean, IQR)	158, 31–290
Hypertension, n (%)	30 (75)
Diabetes, n (%)	17 (42)
Atrial fibrillation, n (%)	12 (31)
COPD, n (%)	2 (5)
CKD, n (%)	15 (37)
OSAS, n (%)	6 (15)
Pharmacologic therapy	
ACE/MRA inhibitors	38 (95)
β-blockers	35 (88)
ARNI	6 (15)

*Abbreviations:* ACE, angiotensin-converting enzyme; ARNI, angiotensin receptor–neprilysin inhibitor; BNP, brain natriuretic peptide; COPD, chronic obstructive pulmonary disease; CKD, chronic kidney disease; HF, heart failure; IQR, interquartile range; MRA, mineralocorticoid receptor antagonist; NYHA, New York Heart Association; OSAS, obstructive sleep apnea syndrome; SD, standard deviation.
